# Leiomyoma of the esophagus: A case report and review of the literature

**DOI:** 10.1016/j.ijscr.2022.107078

**Published:** 2022-04-13

**Authors:** Hazem Beji, Mahdi Bouassida, Yassine Kallel, Mohamed Amine Tormane, Mohamed Mongi Mighri, Hassen Touinsi

**Affiliations:** Department of General Surgery, Hospital Mohamed Taher Maamouri, Nabeul, Tunisia; University Tunis El Manar, Faculty of Medicine of Tunis, Tunisia

**Keywords:** Leiomyoma, Esophagus, Benign tumors, Case report, Literature review

## Abstract

**Introduction:**

Benign tumors represent less than 1% of esophageal neoplasms. Esophageal leiomyoma is a very rare tumor that arises from mesenchymal tissue.

We present here a case of leiomyoma of the esophagus which was revealed by dysphagia and vomiting that was treated by surgical enucleation.

**Presentation of case:**

This report illustrates the case of a female who presented with dysphagia and vomiting. Imageology demonstrated an esophageal mass which was treated with surgical enucleation. Histopathology confirmed the diagnosis of leiomyoma.

**Clinical discussion:**

Benign esophageal tumors are rare. Leiomyoma commonly presents as a single lesion in the middle or lower third of the esophagus. Leiomyomas located in the proximal and middle third of the esophagus can be operated on by right thoracotomy. Surgical treatment varies from enucleation to esophageal resection depending on the size and location of the mass. In our case, the tumor was enucleated by a right posterolateral thoracotomy.

**Conclusion:**

Esophageal leiomyoma is a benign and generally asymptomatic tumor. Surgery is the pillar of treatment. Enucleation should be performed whenever possible to avoid esophagectomy and thus decrease morbidity and mortality.

## Highlights

• Esophageal leiomyoma is a very rare tumor that arises from mesenchymal tissue.
• An enhanced computed tomography scan with oral contrast or endoscopic ultrasonography is essential to assess the diagnosis.
• Surgery consists of enucleation or esophagectomy depending on the location and size of the tumor.

## 1 Introduction and importance

Benign tumors represent less than 1% of esophageal neoplasms. Esophageal leiomyoma is a very rare tumor that arises from mesenchymal tissue. It is the most common benign tumor of the esophagus [1].

More than 50% of the patients are asymptomatic and are diagnosed incidentally while others may present dysphagia or epigastric pain [2].

Surgery is the cornerstone of treatment. It consists of transthoracic extra mucosal blunt enucleation. Esophagectemy is sometimes necessary in presence of giant leiomyomas [3].

We present here a case of leiomyoma of the esophagus which was revealed by dysphagia and vomiting that was treated by surgical enucleation.

This work has been reported in line with the SCARE 2020 criteria [4].

## 2 Presentation of a case

A 47-year-old female, hypertensive, complained of dyspepsia and esophageal reflux.

She presented gradually worsening dysphagia and vomiting six months earlier.

The general physical examination and laboratory findings were normal.

**Fig. 1:**
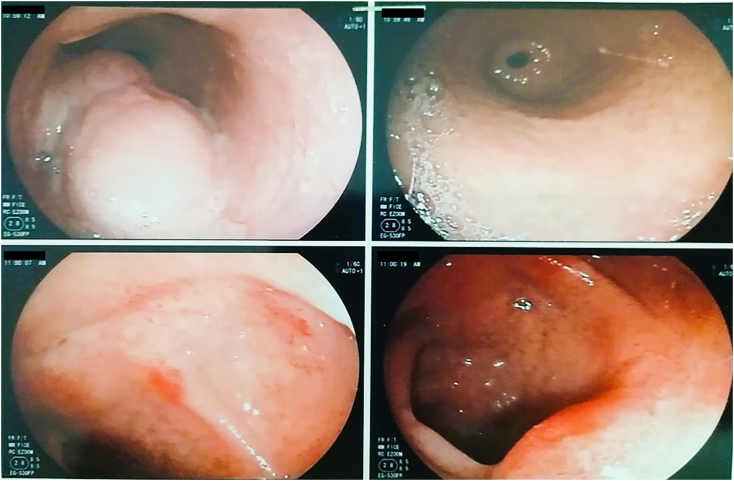
Gastroesophageal fibroscopy revealing compression of the middle third of the esophagus.

A gastroesophageal fibroscopy showed compression of the middle third of the esophagus (Fig. 1).

**Fig. 2:**
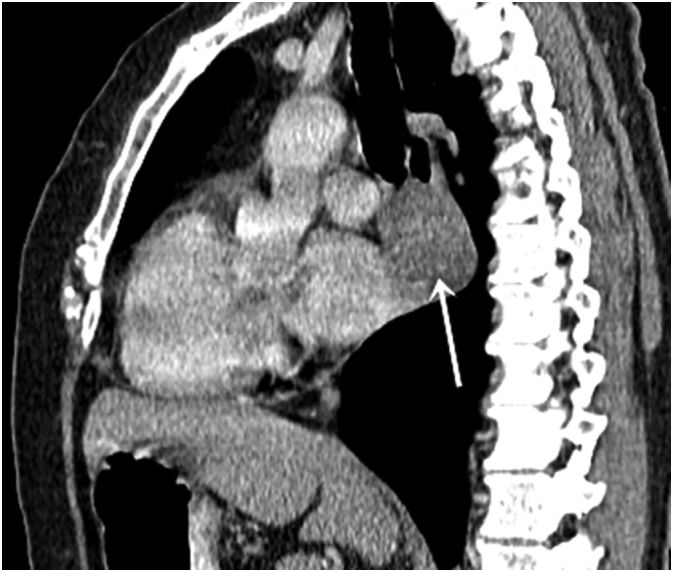
CT scan in the sagittal plane showing a moderately enhancing mediastinal mass displacing the esophagus in its middle third (white arrow).

Abdominal computed tomography (CT) scan revealed a moderately enhancing 30 mm oval posterior mediastinal mass extending over 60 mm, displacing the esophagus in its middle third and coming into contact with the descending aorta with the pericardium (Fig. 2).

**Fig. 3:**
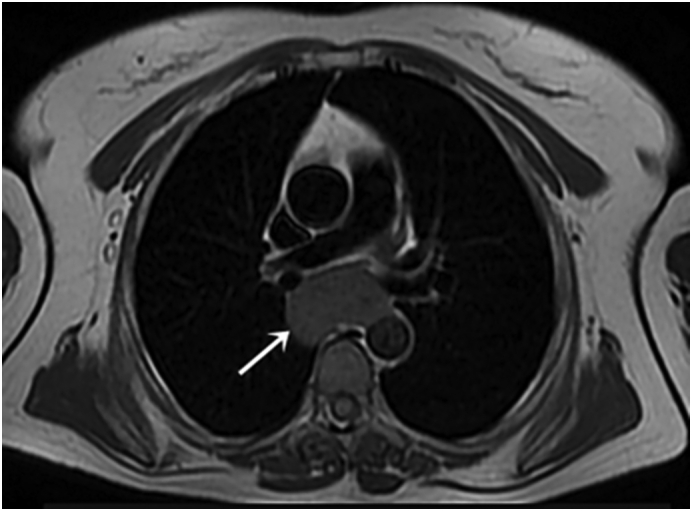
Chest MRI showing esophageal tissular mass (white arrow).

Magnetic resonance imaging (MRI) was showed a tissue mass of esophageal origin under the mucosa presenting contact with the posterior pericardium and with the anterior wall of the aorta over 10 mm (Fig. 3).

Surgery was planned and was performed by a 20-year experience surgeon.

We performed a right posterolateral thoracotomy at the level of the 5th inter-costal space.

**Fig. 4:**
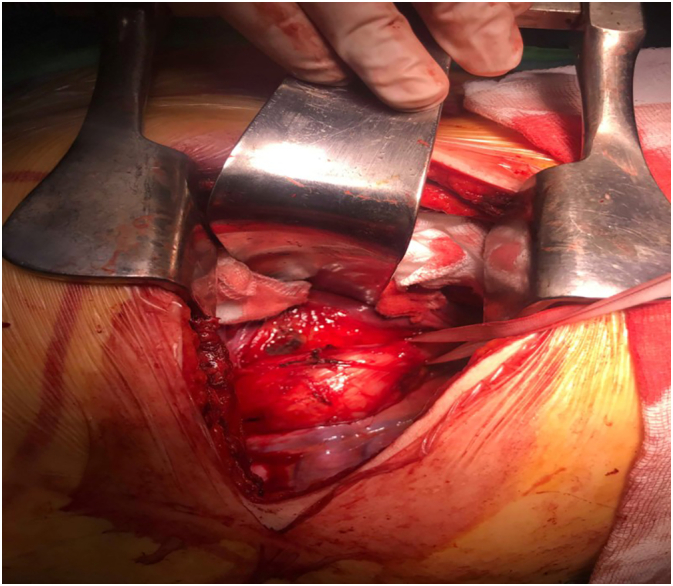
Intraoperative view of the esophageal leiomyoma.

The exploration highlighted a 6 cm hemi-circumferential poly-lobed whitish mass, adherent to the pericardium and the aorta (Fig. 4).

Meticulous dissection was performed all around the esophagus and the tumor was enucleated.

The esophagus has been checked for leaks.

**Fig. 5:**
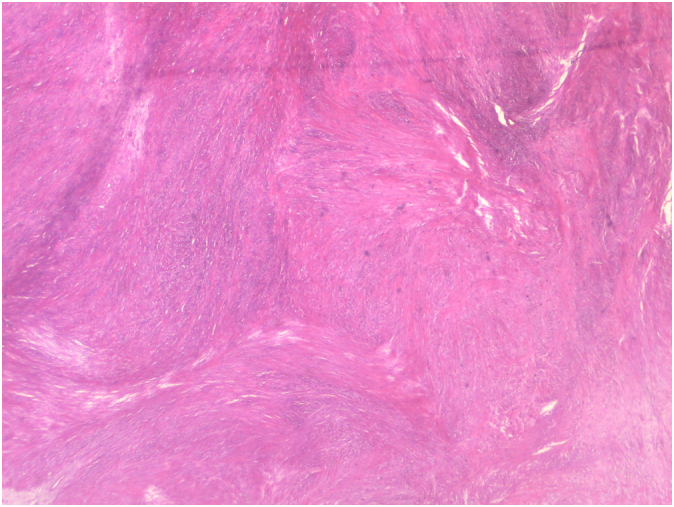
Histopathological slide with hematoxylin and eosin demonstrating neoplastic spindle cells arranged in bundles.

**Fig. 6:**
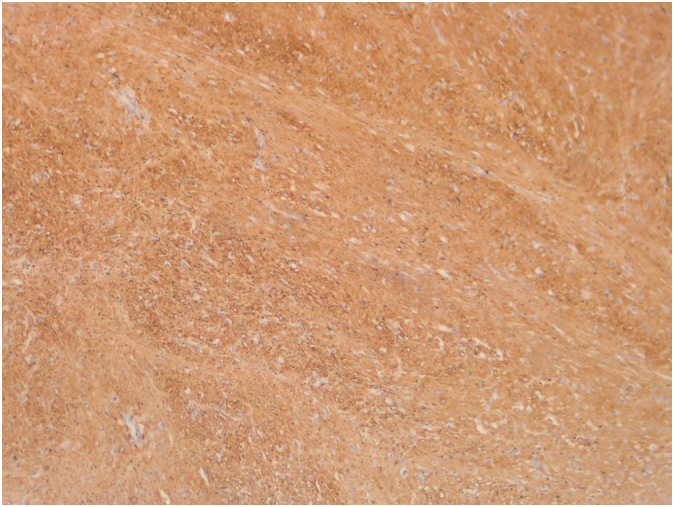
Immunohistochemistry showing positive smooth muscle actin marker.

Histopathology revealed neoplastic spindle cells arranged in bundles without significant cellular atypia (Fig. 5). Immunohistochemical markers were positive for smooth muscle actin (Fig. 6) and negative for CD-117 and Dog-1. The diagnosis of esophageal leiomyoma was confirmed.

The postoperative course was uneventful with a follow-up of eight months.

## 3 Clinical discussion

We reported a successful surgical enucleation for an esophageal leiomyoma. The main strength of our work is the choice for an enucleation avoiding unnecessary esophagectomy and thus decreasing morbidity and mortality.

The main weakness of our work is the choice for a thoracotomy instead of mini-invasive surgery. Our choice is explained by the preoperative imaging showing contact of the tumor with aorta and pericardium.

Benign tumors of the esophagus are rare and represent less than 1% of esophageal neoplasms. Leiomyoma is the most prevalent benign esophageal tumor [1].

These tumors arise from smooth muscle and have an intramural location. In some cases, leiomyoma presents as a pedunculated polyp. Around 60% develop in the distal third of the esophagus [2].

Typically, leiomyomas don't exceed 5 cm. However, giant leiomyomas of more than 20 cm have been reported [5].

Leiomyoma can occur in patients of any age, but the peak incidence is in the third to fifth decades of life. The ratio of cases in men and women is approximately 2:1 [6].

Preoperative diagnosis is difficult as esophageal leiomyoma can present as a posterior mediastinal mass on chest radiograph and can be seen as an incidental radiological finding [2].

Patients are asymptomatic in more than half of the cases [7]. When symptoms are present, they are generally non-specific. Dysphagia and retrosternal pain are found in tumors larger than 5 cm [8]. In our case, dysphagia and vomiting were the main symptoms.

Barium swallow is a widely used imaging technique for the diagnosis of esophageal lesions. It shows smooth filling defects in the esophageal lumen without mucosal damage. The tumor is generally mobile during deglutition [9].

Contrast-enhanced computed tomography (CT) scan with oral contrast is usually sufficient to confirm the diagnosis [10]. Endoscopic ultrasound represents another option showing intramural tumors [11].

It is performed to visualize all layers of the esophageal wall. It can distinguish cystic from solid esophagus tumors. Leiomyoma appears as a homogenous, hypoechoic lesion surrounded by a hyperechoic region [12].

In our case, EUS was not realized. The diagnosis was oriented by a contrast-enhanced CT scan. A magnetic resonance image (MRI) was done to rule out differential diagnoses such as esophageal duplication cyst. It revealed a well-defined soft tissue mass on the posterior wall of the middle third of the esophagus.

The use of the biopsy in the diagnosis is controversial. It is linked to many complications such as infection, bleeding, increased rate of intraoperative perforations, and technical difficulties. It is recommended to perform a biopsy in case of diagnostic doubt [11], [13]. In some cases, needle aspiration biopsy does not accurately identify the nature of the lesion. Therefore, malignancy can only be ruled out by resection [14].

The main differential diagnoses include malignancies such as squamous or adenomatous carcinoma and leiomyosarcoma. Though, malignant transformation is very rare [7].

There are no clear therapeutic guidelines. Some authors recommend resection to rule out malignancy in all cases [11], [13], [15]. Other authors recommend observation for the asymptomatic patient with lesions less than 5 cm in size and when the preoperative workup has eliminated malignancy [2]. In those cases, a regular follow-up with barium swallow is essential [16]. Regular follow-up of asymptomatic patients with a tumor size of less than 2 cm has been promoted [17].

Minimally invasive surgery is a good option in tumors less than 5 cm [18]. Surgical treatments by thoracoscopy [11], laparoscopy [18], or Da Vinci robot-assisted thoracoscopy [19] were reported.

The standard surgical approach for esophageal leiomyoma is a thoracotomy with enucleation without opening the mucosa which is easier, faster, and safer compared to resection [17]. It is indicated for tumors in the proximal and middle third of the esophagus [15].

A trans-hiatal approach can be performed for tumors located in the lower third of the esophagus [2].

An esophageal resection is appropriate for large tumors and those located at the gastroesophageal junction due to technical difficulties, poor wound healing in the defect of esophageal muscle, and lower esophageal sphincter dysfunction or loss following enucleation [20].

In our case, the contact of the tumor with the pericardium and aorta made us opt for a thoracotomy.

In summary, we reported the case of a 47-year-old hypertensive patient complaining of dysphagia and vomiting due to a 6 cm leiomyoma in the middle third of the esophagus diagnosed by a CT scan and MRI. A successful surgical enucleation by a right posterolateral thoracotomy was performed.

## 4 Conclusion

Esophageal leiomyoma is a slowly growing benign and asymptomatic tumor. The symptoms appear when the tumor is large. The diagnosis is confirmed with a contrast-enhanced CT scan and EUS with biopsies.

Surgery is the pillar of treatment. Tumor enucleation or esophageal resection can be performed depending on the size and location of the mass.

## Patient consent

Written informed consent was obtained from the patient for publication of this case report and accompanying images. A copy of the written consent is available for review by the Editor-in-Chief of this journal on request.

## Provenance and peer review

Not commissioned, externally peer-reviewed.

## Ethical approval

Ethical approval for this study was obtained from the ethical committee of the hospital Mohamed Taher Mammouri.

## Funding

This research did not receive any specific grant from funding agencies in the public, commercial, or not-for-profit sectors.

## Guarantor

Hazem Beji
Mahdi Bouassida.

## Research registration number

None.

## CRediT authorship contribution statement

Hazem Beji and Mahdi Bouassida did the conception and design of the work, the data collection, and the data analysis and interpretation.

Yassine Kallel and Mohamed Amine Tormane did the critical revision of the article.

Mohamed Mongi Mighri and Hassen Touinsi did the final approval of the version to be published.

## Declaration of competing interest

No conflicts of interest.
